# Bioinformatics Identification of Aberrantly Methylated Differentially Expressed Genes Associated with Arteriosclerosis by Integrative Analysis of Gene Expression and DNA Methylation Datasets

**DOI:** 10.3390/genes13101818

**Published:** 2022-10-08

**Authors:** Jin Cheng, Yuli Hou, Cong Wang, Lianrui Guo

**Affiliations:** 1 Department of Vascular Surgery, Xuanwu Hospital, Capital Medical University, Beijing 100053, China; 2 Clinical Laboratory of Xuanwu Hospital, Capital Medical University, Beijing 100053, China

**Keywords:** arteriosclerosis, methylation, Gene Expression Omnibus, vascular smooth muscle cells, LMOD1

## Abstract

The prognosis of patients with advanced arteriosclerosis is bleak due to the lack of understanding of arteriosclerosis. Epigenetics-based DNA methylation plays an important role in the pathogenesis of arteriosclerosis. Hence, we aimed to identify the epigenetics-related aberrantly methylated differentially expressed genes (AMDEGs) in arteriosclerosis. A gene expression dataset and DNA methylation dataset were downloaded from the Gene Expression Omnibus database, and AMDEGs were identified on the basis of the relationship between methylation and expression. Subsequently, the expression levels of candidate hub genes were detected in human peripheral blood mononuclear cells (PBMCs) from atherosclerotic patients and control subjects by RT-qPCR and Western blot. Lastly, the methylation level of the target gene was detected using the MassARRAY method. In the present study, the hypermethylated and downregulated genes were mainly involved in vascular smooth muscle contraction. The hypomethylated and upregulated genes were markedly associated with immune-inflammatory processes. Following validation, LMOD1 was identified as the target gene, which was hypermethylated and downregulated in arteriosclerosis. The methylation levels of CpG sites in LMOD1 promoter were detected to be elevated in the PBMCs of atherosclerotic patients. In conclusion, AMDEGs identified in the present study may assist in understanding the pathogenesis of arteriosclerosis. LMOD1 exhibits potential as a promising diagnostic and therapeutic biomarker for arteriosclerosis.

## 1. Introduction

Despite the markedly reduced mortality of atherosclerotic cardiovascular disease as a result of the application of new therapies, arteriosclerosis (AS) is still the primary cause of mortality worldwide [1]. Prognosis is bleak for patients with AS involving vital organs. Therefore, it is necessary to seek out effective biomarkers for early detection and preventive treatment. Recently, the role of epigenetics in identifying the pathogenesis of AS has been approved [2]. Epigenetics is characterized as the study of any underlying alterations in cellular phenotype and gene expression that happens without alterations in Watson–Crick base-pairing of the DNA sequence. Epigenetic mechanisms include DNA methylation, histone acetylation, and noncoding RNA regulation. DNA methylation, as an important regulatory mechanism of gene transcription, is a stable and heritable epigenetic form. Genomes and the environment interact at the epigenetic interface, in a manner such that environmental factors can alter the DNA methylome to alter gene expression patterns without alterations in the underlying genomic sequence. DNA methylation/demethylation marks the influence of environmental factors on gene expression; thus, it may provide a potential explanation for disease discordance despite genetic similarities. Therefore, in recent studies, genome-wide gene expression (transcriptomics) and DNA methylation (epigenomics) were comprehensively analyzed to identify biomarkers of environmental influence on diseases. Various diseases have been verified to be associated with aberrant DNA methylation. Eight cancer-related hypermethylated gene markers were identified in clinical guidelines [3]. DNA methylation modification is positively correlated with the progress of atherosclerotic patients with coronary heart disease [4]. Human and mouse studies have revealed that DNA hypermethylation is an accompanying feature of AS [5,6]. Recent studies showed a special methylation profile in atherosclerotic patients and suggested that many factors, including inflammation, hyper-homocysteinemia, shear stress, low-density lipoprotein oxidation, and aging, can stimulate methylation in AS [7,8,9,10]. Similar to clinical application of DNA-hypomethylating drugs in leukemia, targeting specific alterations in DNA methylation may represent promising new treatment possibilities for AS. Nevertheless, systematic analysis of the effect of DNA methylation on AS is still lacking. Exploring AS-related methylated genes and their mechanisms and functions will help to identify new biomarkers for AS. Since the 21st century, the increasing use of bioinformatics technology has helped us to seek out the underlying genetic targets of diseases, helping researchers to identify the genes and potential pathways that may be related to the occurrence and progress of AS.

It is well known that the carotid plaque is associated with an increased risk of stroke and cerebral infarction, and the presence of carotid plaque mirrors different degrees of AS and conveys a higher risk [11,12,13]. In addition, carotid plaque is available from patients undergoing endarterectomy. Thus, carotid atherosclerotic samples are widely used for identifying atherosclerotic diseases.

In this study, we downloaded and analyzed the gene expression profile and DNA methylation profile from the Gene Expression Omnibus (GEO) database. We obtained the aberrantly methylated differentially expressed genes (AMDEGs) between atheroma plaques and healthy control artery samples. We further analyzed the biological functions of AMDEGs and obtained hub genes via protein–protein interaction (PPI) network analysis. In addition, we validated the expression and methylation levels of hub genes with other GEO datasets and human peripheral blood samples.

## 2. Materials and Methods

### 2.1. Microarray Data Profile

**Figure 1:**
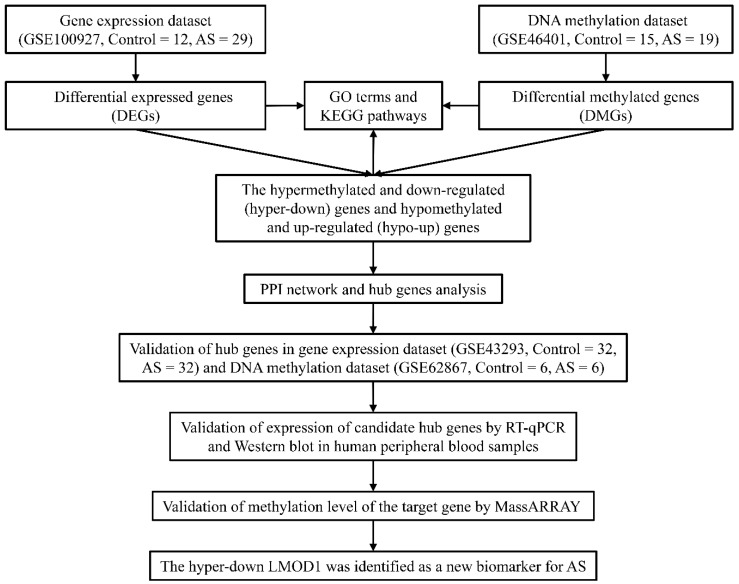
Flowchart of the analysis process.

Gene Expression Omnibus (GEO) is an online database that provides comprehensive data on gene profiling and sequencing. In GEO database, we retrieved the gene expression profile dataset GSE100927 along with DNA methylation profile dataset GSE46401. The dataset GSE100927, including 12 normal control carotid artery samples and 29 carotid atherosclerotic samples, was based on the GPL17077 platform Agilent-039494 SurePrint G3 Human GE v2 8 × 60 k Microarray. The dataset GSE46401, containing 19 carotid atherosclerotic samples and 15 control aortic tissue, was assayed on the platform of GPL13534 [Human Methylation450_15017482] Illumina HumanMethylation450 BeadChip covering approximately 450,000 5′-C–phosphate–G-3′ (CpG) sites at different gene regions containing TSS1500, TSS200, 5′UTR, 1stExon, body, and 3′UTR. TSS1500 refers to 200–1500 bases upstream of the transcriptional start site (TSS). TSS200 refers to 0–200 bases upstream of TSS. 5′UTR stands for the 5′ untranslated region, defined as the region between the TSS and the ATG start site. 1stExon is short for the first exon of the gene. Body is the region between ATG start site and stop codon. 3′UTR is short for the 3′ untranslated region that is between the stop codon and poly-A tail. The workflow of this study is shown in Figure 1.

### 2.2. Data Processing and Identification of DEGs and DMGs

First, principal component analysis (PCA) was performed to determine the sample distribution using the R “gmodels” package. For the identification of differentially expressed genes (DEGs) and differentially methylated genes (DMGs), the R “limma” package was applied to process GSE100927, and the R “ChAMP” package was used to process the GSE46401 dataset. The DEGs were identified with logFC > 1 and adj-*p*-value < 0.05, and DMGs were screened with criteria logFC > 0.2. The R “ggplot” package was applied to draw heat maps and volcano plots of the DEGs and DMGs.

### 2.3. Integrative Analysis of DNA Methylation and Gene Expression

The methylation-mediated genes in AS were further screened by integrative analysis of DMGs and DEGs. A Venn diagram was completed in FunRich software to identify hypomethylated and upregulated (hypo-up) genes and hypermethylated and downregulated (hyper-down) genes.

### 2.4. Functional Enrichment Analysis

For further investigation of underlying molecular mechanisms of the dysregulated genes, the R “clusterProfiler” package was used for Gene Ontology (GO) annotation and Kyoto Encyclopedia of Genes and Genomes (KEGG) pathway enrichment analysis to discover the potential biological functions of the DEGs, DMGs, and AMDEGs. A *p*-value < 0.05 was considered statistically significant. GO annotation analysis includes three sections: biological process (BP), molecular function (MF), and cellular component (CC).

### 2.5. Protein–Protein Interaction (PPI) Network Construction and Hub Gene Identification

Using the search tool for the retrieval of interacting genes/proteins (STRING) online database (http://string-db.org/, accessed on 1 May 2020), the PPI networks for hyper-down genes and hypo-up genes were built, and the PPI score was set as 0.4. Then, PPI networks were visualized using Cytoscape software (supported by NIGSM & NRNB, USA). Genes served as “nodes” in the PPI network, and the line segment between two nodes represented interactions. “CytoHubba” was used to determine the hub genes.

### 2.6. Validation of the Hub Genes with other GEO Datasets

We validated the expression and methylation levels of hub genes with other datasets. The gene expression dataset GSE43292, containing 32 paired macroscopically intact tissues and atheroma plaque tissues, was used to validate the expression levels of the hub genes. The gene methylation levels of hub genes were validated with DNA methylation dataset GSE62867, including six paired internal mammary artery tissue and coronary atherosclerotic plaques. The statistical significance was performed using paired t-tests with GraphPad Prism software (San Diego, CA, USA) when comparing the two groups, and *p* < 0.05 was considered statistically significant.

### 2.7. Validation of Candidate Hub Genes with Human Peripheral Blood Samples

#### 2.7.1. Peripheral Blood Mononuclear Cells Isolation

Healthy volunteers without cancer, blood diseases, AS, diabetes, and acute or chronic inflammation were recruited from the health screening center of Xuanwu Hospital. Blood with the anticoagulant EDTA-K2 was collected from healthy volunteers (*n* = 20) and atherosclerotic patients (*n* = 20) who signed informed consent forms. The two populations were matched by age and gender. Peripheral blood mononuclear cells (PBMCs) were obtained using Ficoll-Paque (Solarbio, Beijing, China) density gradient centrifugation. The use of human specimens was approved by appropriate institutional review boards.

#### 2.7.2. Quantitative Reverse Transcription Polymerase Chain Reaction (RT-qPCR)

Total RNA was isolated from PBMCs with TRIzoL reagent (Thermo Fisher, Rockford, IL, USA) and then reversed. RT-qPCR was performed using SYBR (Takara, Japan) on a Roche 480 machine (Basel, Switzerland). Quantification was performed using the 2^−ΔΔCT^ method to calculate mRNA relative expression levels. The primer sequences for PCR amplification of candidate hub genes are listed in Table 1. 

#### 2.7.3. Western Blot Analysis

Total cellular protein was extracted by adding RIPA (Solarbio, Beijing, China) assay buffer containing protease inhibitors (Solarbio, Beijing, China). The protein concentration was determined using a BCA Protein Assay kit (Thermo Fisher Scientific, Rockford, IL, USA). Equal amounts of protein were loaded for SDS-PAGE electrophoresis. Then, the proteins were transferred from the gels to PVDF membranes (Millipore, Billerica, MA, USA). The membranes were blocked by immersing in 5% nonfat milk in TBST for 1 h to inhibit nonspecific binding before being incubated with primary antibodies LMOD1 (1:1000, Abcam, Cambridge, UK), TLR1 (1:1000, Abcam, Cambridge, UK), C1QA (1:1000, Abcam, Cambridge, UK), and β-actin (1:5000, Zhongshan Boil Tech Co., Beijing, China) at 4 °C overnight.

### 2.8. Quantification of DNA Methylation

Genomic DNA was extracted and bisulfite-converted from the peripheral blood of healthy donors and atherosclerotic patients using a genomic DNA extraction kit (BioTeKe Corpration, Beijing, China). The quantification of DNA methylation was detected using the quantitative high-resolution mass spectrometry-based approach (MassARRAY) platform (Agena Bioscience, San Diego, CA, USA) by BGI Tech Solutions Co. (Shanghai, China). As a prediction, 15 CpG sites were located at the LMOD1 promoter region starting from −520 to −56 (464 bp), but five of them were undetectable. The sequences for primers were designed using the Sequenom EpiDesigner software (forward: 5′-TTTGGTAGAATTTGGGTAGAAAGAA-3′ and reverse: 5′-AAAACCAATCCCCAAACTAAAAAC-3′). The test results were obtained using Agena’s EpiTYPER™ software (San Diego, CA, USA).

### 2.9. Statistical Analysis

All statistical analysis was performed using R software version 3.6.3 (The R Foundation for Statistical Computing, Vienna, Austria) and GraphPad Prism 9 (San Diego, CA, USA). Experimental statistics were represented as means ± SD. The *t*-test was used to compare the difference between the two groups. A *p*-value < 0.05 was considered statistically significant.

## 3. Results

### 3.1. Identification of DEGs and DMGs in Human Atherosclerotic Plaques

**Figure 2:**
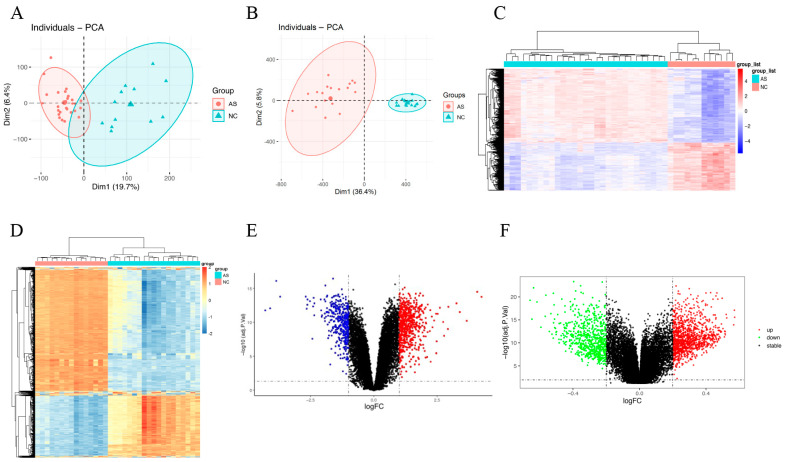
Identification of differentially expressed genes (DEGs) in gene expression profile dataset GSE100927 and differentially methylated genes (DMGs) in DNA methylation profile dataset GSE46401 between normal control arteries (NC) and arteriosclerotic lesions (AS). The cutoff standards for DEGs and DMGs were logFC > 1, *p* < 0.05 and logFC > 0.2, *p* < 0.05, respectively. (**A**) Principal component analysis (PCA) of the sample distribution in GSE100927. (**B**) PCA of the sample distribution in GSE46401. (**C**) Heatmap of DEGs identified in GSE100927. (**D**) Heatmap of DMGs identified in GSE46401. (**E**) Volcano plot of DEGs in GSE100927. (**F**) Volcano plot of DMGs in GSE46401.

Principal component analysis (PCA) was visualized to show an obvious distribution difference between atherosclerotic lesions and normal control artery samples in transcriptome profile in GSE100927 (Figure 2A) and methylation profile in GSE46401 (Figure 2B). Following the screening of the objective dataset for gene differential expression analysis, we obtained 994 DEGs, including 391 downregulated genes and 603 upregulated genes. On the other hand, a total of 5817 DMGs were obtained from GSE46401 dataset, including 3081 hypermethylated genes and 2736 hypomethylated genes. On the basis of the gene expression levels in the GSE100927 dataset and DNA methylation levels in the GSE46401 dataset, we generated heat maps (Figure 2C,D) and volcano maps (Figure 2E,F) to show the DEGs and DMGs, helping us significantly discriminate the DEGs and DMGs between AS samples and normal samples.

### 3.2. Identification of DMGs in AS Based on Different Region CpGs

**Figure 3:**
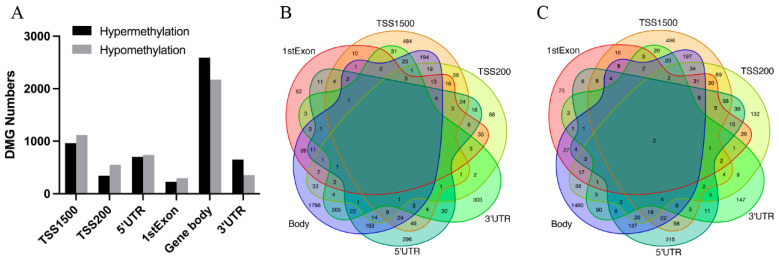
Integration analysis results of DMGs based on different region CpGs. (**A**) Bar plot of hypermethylated DMGs and hypomethylated DMGs in each region. Venn plot for (**B**) hypermethylated DMGs and (**C**) hypomethylated DMGs in different regions. The numbers on the diagram represent the DMG numbers in a specific region or multiple regions. Each region name is labeled beside the region circle.

CpG sites were distributed at different gene regions including TSS1500, TSS200, 5′UTR, 1stExon, body, and 3′UTR. As Figure 3A shows, there were 523 DMGs with a CpG site in the 1stExon region, 1006 DMGs with a CpG site in the 3′UTR region, 1439 DMGs with a CpG site in the 5′UTR, 2080 DMGs with a CpG site at TSS1500, 894 DMGs with a CpG site at TSS200, and 4765 DMGs with a CpG site in the body region. The CpG site distribution in different regions of hypermethylated genes and hypomethylated genes was similar. Nearly 50% of methylation alterations occurred in the body region. DMGs were mostly shared among different regions, while more than 50% of DMGs in the body region were region-specific DMGs (Figure 3B,C). These results revealed that methylation alterations of DMGs in AS occurred at multiple CpG sites in the gene sequence. In addition, two hypomethylated genes (HOXD3 and HOXD4) were found to be shared among all regions, which encode a highly conserved family of transcription factors and play an important role in morphogenesis in all multicellular organisms. 

### 3.3. The Identification and CpG Sites Distribution of AMDEGs

**Figure 4:**
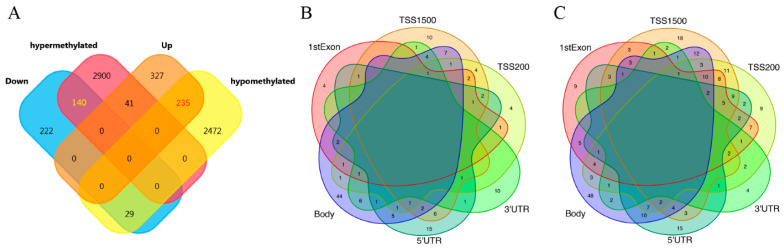
Integration analysis of aberrantly methylated-differentially expressed genes (AMDEGs) and distribution of AMDEGs based on different region CpGs. (**A**) Venn diagram showing the overlapping aberrantly methylated differentially expressed genes (AMDEGs). Venn plot for (**B**) hyper-down genes and (**C**) hypo-up genes in AS in different regions. Hyper-down genes represent hypermethylated and downregulated genes. Hypo-up genes represent hypomethylated and upregulated genes.

The methylation microarray dataset and gene expression microarray dataset were integrated and analyzed to identify the AMDEGs. As shown in Figure 4A, 140 hypermethylated and downregulated (hyper-down) genes, 41 hypermethylated and upregulated (hyper-up) genes, 29 hypomethylated and downregulated (hypo-down) genes, and 235 hypomethylated and upregulated (hypo-up) genes were identified. The distribution of CpG sites of hyper-down genes according to gene regions was as follows: 1stExon (14 genes), 3′UTR (26 genes), 5′UTR (40 genes), Body (78 genes), TSS1500 (44 genes), and TSS200 (22 genes) (Figure 4B). The CpG sites were distributed at different regions of hypo-up genes, including 1stExon (70 genes), 3′UTR (18 genes), 5′UTR (68 genes), body (120 genes), TSS1500 (105 genes), and TSS200 (93 genes) (Figure 4C). The results indicated that the CpG sites of AMDEGs were mainly distributed in the body region, especially in hyper-down genes. The CpG sites of hyper-down genes had the lowest number in the 1stExon region, while the CpG sites of hypo-up genes had the lowest number in the 3′UTR region.

### 3.4. DEG Enrichment Analysis

**Figure 5:**
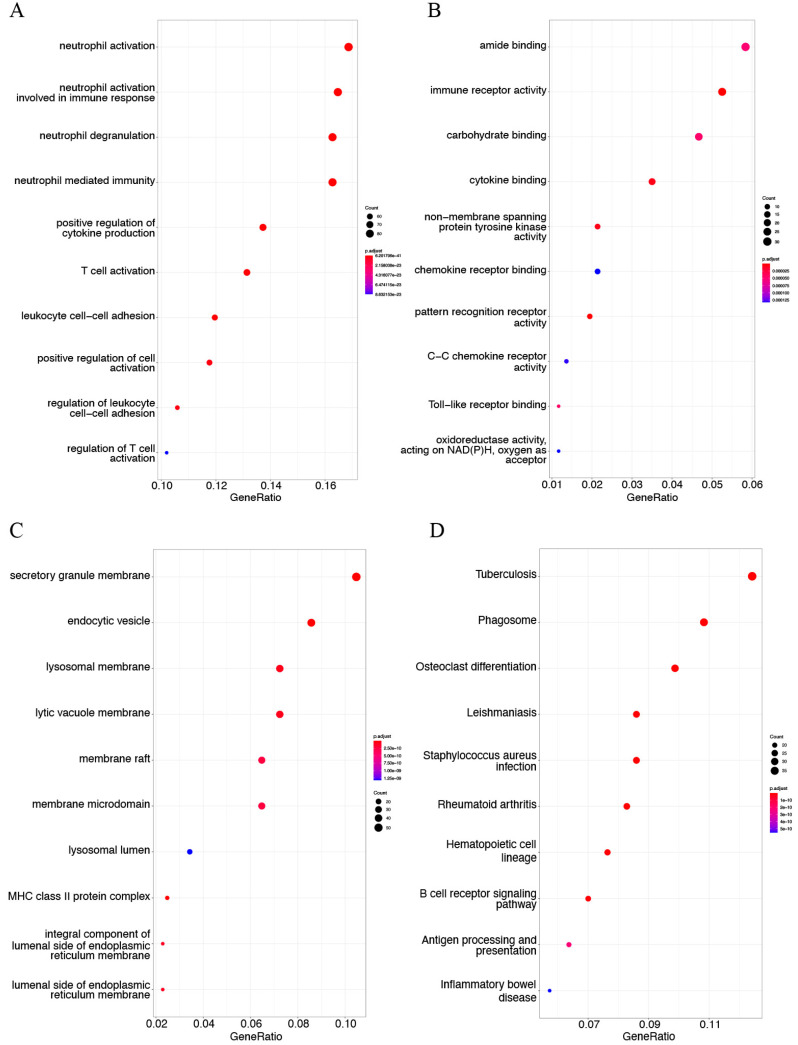
Gene Ontology (GO) term annotation and Kyoto Encyclopedia of Genes and Genomes (KEGG) pathway analysis of upregulated DEGs in AS in GSE100927. Detailed information relating to changes in the (**A**) biological processes (BP), (**B**) molecular functions (MF), and (**C**) cellular components (CC) of upregulated DEGs between control samples and atherosclerotic tissues through GO enrichment analysis. (**D**) The KEGG pathway analysis of upregulated DEGs.

**Figure 6:**
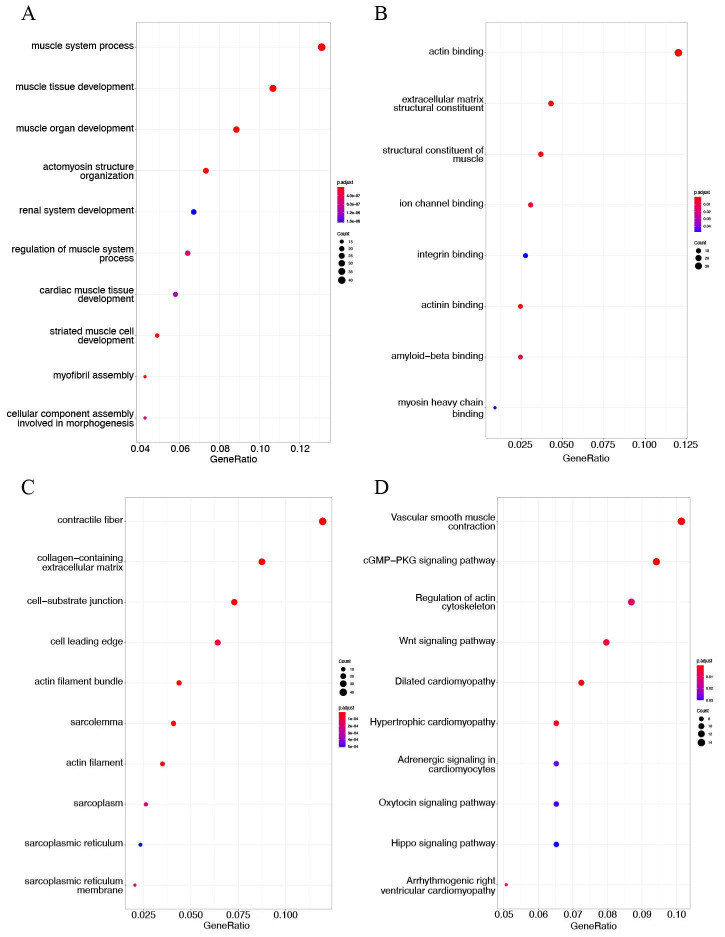
GO term enrichment and KEGG pathway analysis of downregulated DEGs in AS in GSE100927. GO term annotation analysis includes three sections: (**A**) biological process (BP), (**B**) molecular function (MF), (**C**) and cellular component (CC). (**D**) The KEGG pathway analysis of downregulated DEGs.

The top 10 enriched GO terms and KEGG pathways of both upregulated DEGs and downregulated DEGs are listed in Figure 5 and Figure 6, respectively. The results revealed that the upregulated DEGs were mainly involved in the immune-inflammatory response, including neutrophil activation in BP terms (Figure 5A), immune receptor activity and cytokine binding in MF terms (Figure 5B), and secretory granule membrane and endocytic vesicle in CC terms (Figure 5C). KEGG pathway analysis showed that the upregulated genes were markedly related to tuberculosis, phagosome, and osteoclast differentiation (Figure 5D). The GO term analysis found that downregulated genes were mainly enriched in vascular smooth muscle contractility, including muscle process and development in BP terms (Figure 6A), actin-binding in MF terms (Figure 6B), and contractile fiber in CC terms (Figure 6C). These findings were consistent with KEGG enrichment results showing that downregulated genes were mainly associated with vascular smooth muscle contraction (Figure 6D). Furthermore, the downregulated DEGs were also enriched in cGMP-PKG signaling pathway, Wnt signaling pathway, and regulation of actin cytoskeleton in KEGG pathway enrichment. 

### 3.5. DMG Enrichment Analysis

**Figure 7:**
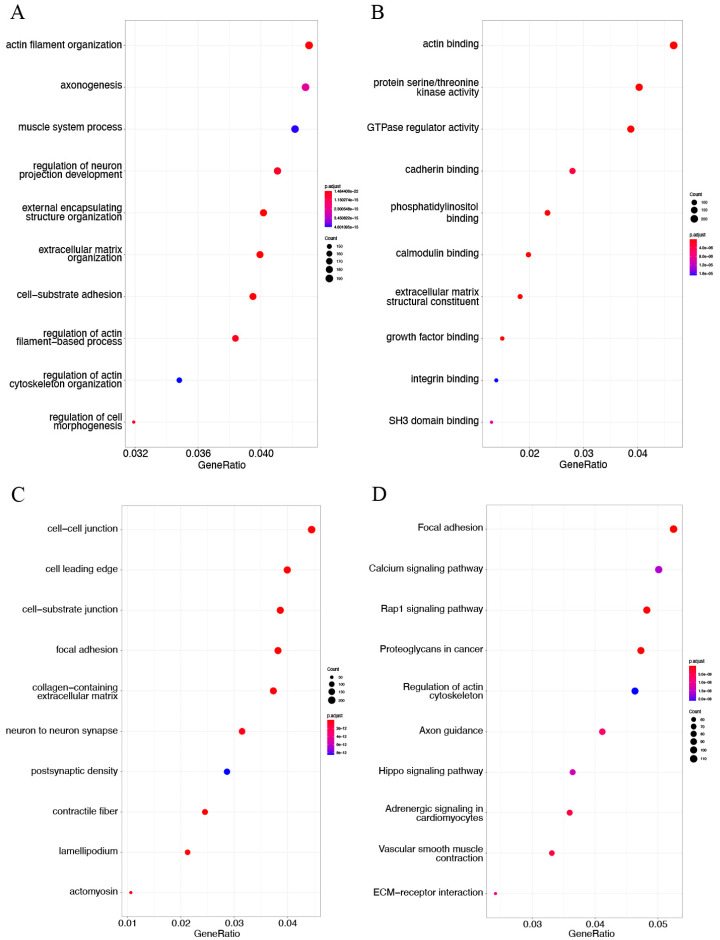
GO term annotation and KEGG pathway analysis of hypermethylated DMGs in AS in GSE46401. Detailed information relating to changes in the (**A**) biological processes (BP), (**B**) molecular functions (MF), and (**C**) cellular components (CC) of hypermethylated DMGs between control samples and atherosclerotic tissues through GO enrichment analysis. (**D**) KEGG pathway analysis of hypermethylated DMGs.

**Figure 8:**
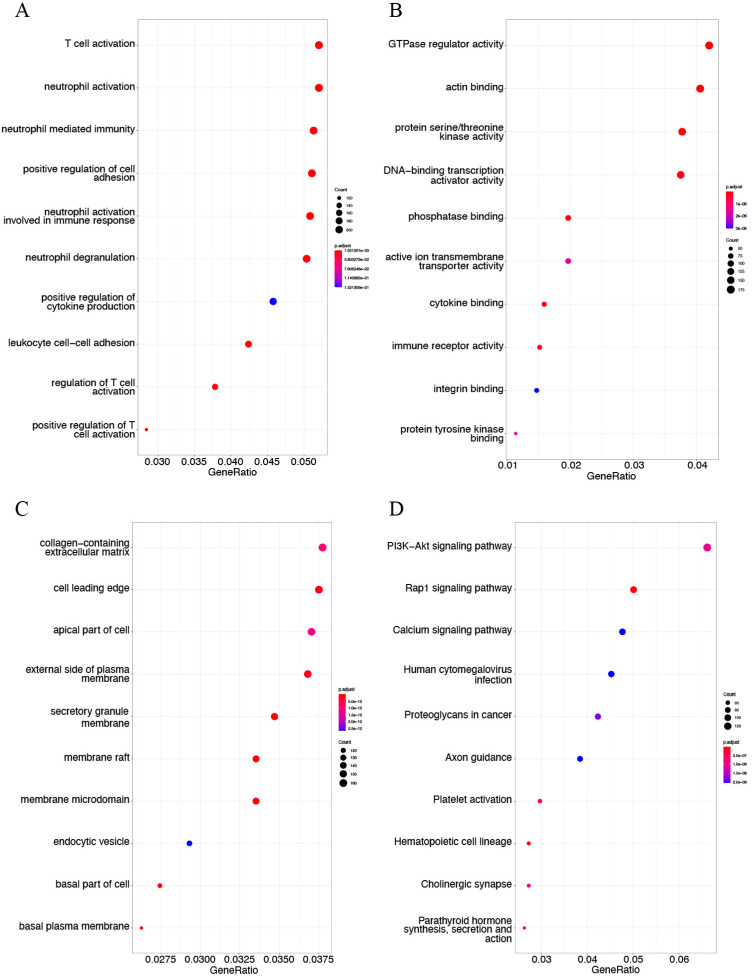
GO term enrichment and KEGG pathway analysis of hypomethylated DMGs in AS in GSE46401. GO term enrichment analysis includes three sections: (**A**) biological process (BP), (**B**) molecular function (MF), and (**C**) cellular component (CC). (**D**) The KEGG pathway analysis of hypomethylated DMGs.

The GO term enrichment and KEGG pathway analyses were performed with 3081 hypermethylated genes and 2736 hypomethylated genes. The top enriched GO terms of hypermethylated genes were actin filament organization in BP terms (Figure 7A), actin-binding in MF terms (Figure 7B), and cell–cell junction in CC terms (Figure 7C). KEGG analysis results revealed that hypermethylated genes were mainly related to the focal adhesion pathway, Rap1 signaling pathway, proteoglycans in cancer, and vascular smooth muscle contraction (Figure 7D). The hypomethylated genes were significantly associated with the activation of neutrophils and T cells in GO BP terms (Figure 8A). In GO MF terms, hypomethylated genes were mainly linked to GTPase regulator activity, actin-binding, protein serine/threonine kinase activity, and DNA-binding transcription activator activity (Figure 8B). Hypomethylated genes were markedly involved in cell leading edge and the biological behavior of membrane structure in GO CC terms (Figure 8C). The results of KEGG analysis found that hypomethylated genes were mainly associated with the Rap1 signaling pathway and PI3K/Akt signaling pathway (Figure 8D).

### 3.6. AMDEG Enrichment Analysis

**Figure 9:**
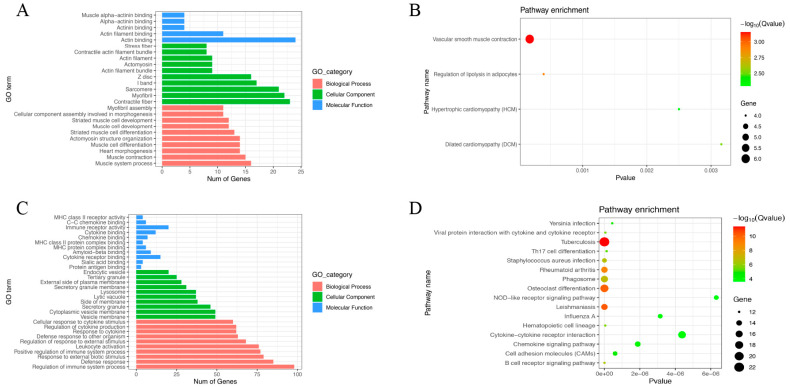
GO and KEGG enrichment analysis of hyper-down genes and hypo-up genes in AS. (**A**) GO term annotation analysis of hyper-down genes. (**B**) KEGG pathway analysis of hyper-down genes. (**C**) GO term enrichment analysis of hypo-up genes. (**D**) KEGG pathway analysis of hypo-up genes.

GO term analysis was performed on hyper-down genes and hypo-up genes, and the top 10 terms are listed. As for the GO BP terms, hyper-down genes were mainly associated with muscle-related biological processes, including muscle system process, muscle contraction, heart morphogenesis, actomyosin structure organization, muscle cell development and differentiation, and myofibril assembly. The hyper-down genes were markedly related to actin-binding in GO MF terms. The GO CC terms of hyper-down genes were mostly enriched in contractile fiber, myofibril, and actin filament (Figure 9A). The results of KEGG pathway enrichment showed that most hyper-down genes were involved in vascular smooth muscle contraction, and a few hyper-down genes were associated with the regulation of lipolysis in adipocytes, hypertrophic cardiomyopathy, and dilated cardiomyopathy (Figure 9B). On the other hand, the GO BP terms of hypo-up genes were mainly enriched in immune processes, defense response, response to external stimulus, leukocyte activation, and cytokine response. As for GO MF terms, these hypo-up genes were markedly related to immune receptor activity, cytokine binding, chemokine binding, and MHC protein complex binding. The GO CC terms of hypo-up genes were significantly enriched in vesicle membrane and secretory granule (Figure 9C). KEGG analysis indicated that hypo-up genes were mainly involved in tuberculosis, osteoclast differentiation, leishmaniasis, rheumatoid arthritis, phagosome, and cytokine–cytokine receptor interaction (Figure 9D).

### 3.7. PPI Network Analysis and Hub Genes Identification

**Figure 10:**
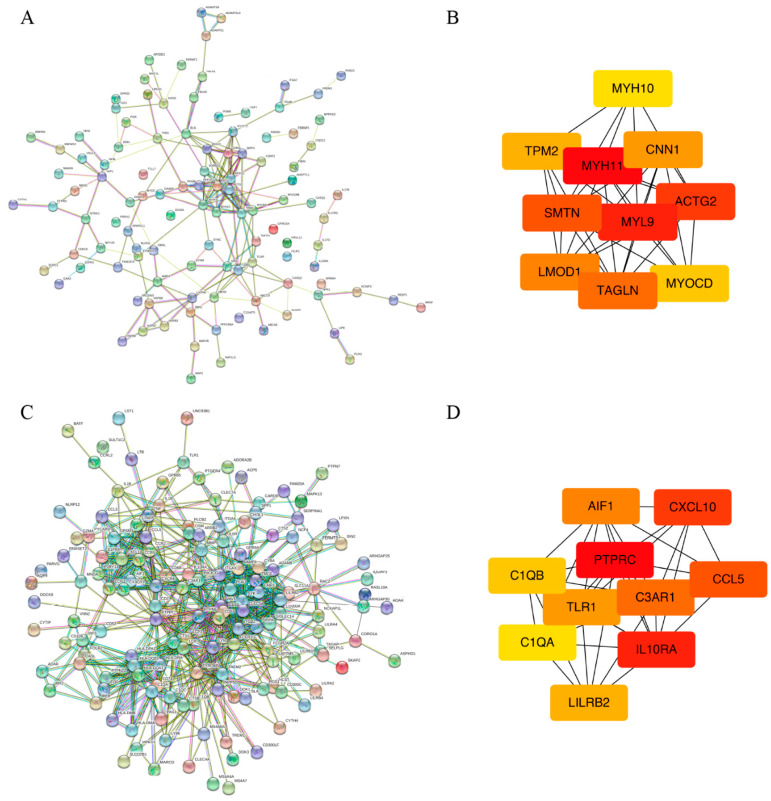
PPI network analysis and hub gene selection of hyper-down genes and hypo-up genes in AS using gene expression dataset (GSE100927) and DNA methylation dataset (GSE46401). The top 10 hyper-down/hypo-up genes with the highest degree of connectivity were determined as hub genes, which were selected using the Maximal Clique Centrality (MCC) method through the CytoHubba plugin of Cytoscape. (**A**) The protein–protein interaction (PPI) network of hyper-down genes. (**B**) The hub genes of hyper-down genes. (**C**) The PPI network of hypo-up genes. (**D**) The hub genes of hypo-up genes. Hyper-down genes represent hypermethylated and downregulated genes. Hypo-up genes represent hypomethylated and upregulated genes. Line color indicates the type of interaction evidence; blue lines represent gene co-occurrence, black lines represent co-expression, and pink lines represent experiment determined relations. PPI enrichment *p*-value < 1.0 × 10^−16^.

The STRING database was used to analyze PPI networks of AMDEGs, and we visualized the results using Cytoscape software. A total of 51 nodes and 37 edges were involved in the PPI network of the hyper-down genes (Figure 10A). Meanwhile, the top 10 hyper-down genes with the highest degree of connectivity were determined as hub genes in the present study. The color changed from yellow to red was indicative of the rank of protein, and a deeper red staining indicated a higher rank of protein. The results revealed that MYH11 was the most significant gene with the greatest connectivity degree, followed by MYL9, ACTG2, SMTN, TAGLN, LMOD1, CNN1, TPM2, MYOCD, and MYH10 (Figure 10B). The PPI network of the hypo-up genes contained 55 nodes and 31 edges (Figure 10C). Furthermore, the PTPRC was identified as the most significant gene with the greatest connectivity degree, followed by IL10RA, CXCL10, CCL5, C3AR1, AIF1, TLR1, LILRB2, C1QB and C1QA (Figure 10D). 

### 3.8. Validation of the Hub Genes in other Datasets

**Figure 11:**
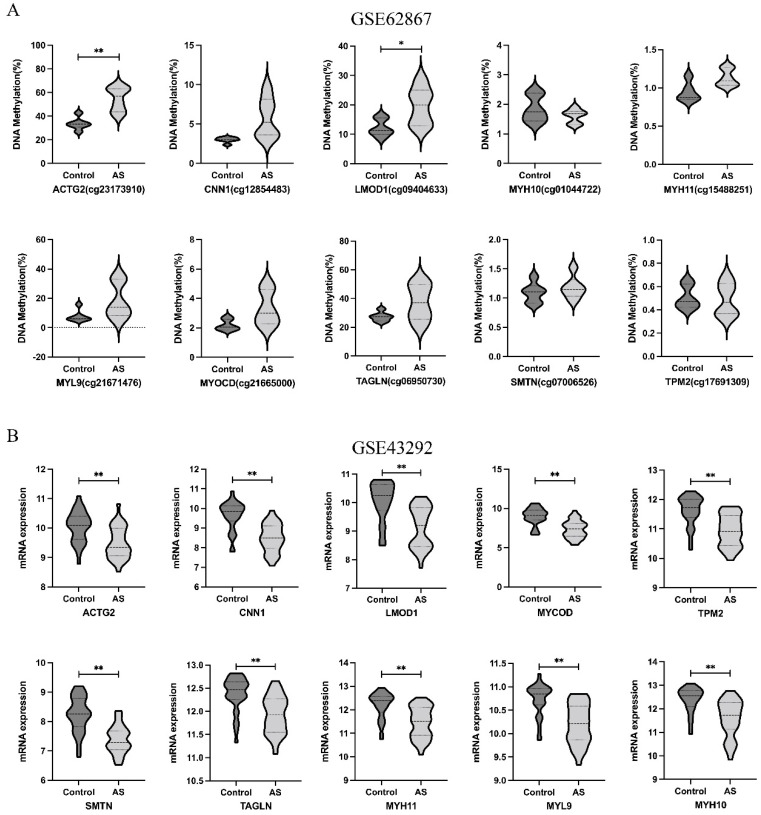
Validation of expression levels and methylation levels of hyper-down genes in AS in gene expression dataset (GSE43292) and DNA methylation dataset (GSE62867). (**A**) The methylation levels of hyper-down genes in GSE62867. (**B**) The expression levels of hyper-down genes in GSE43292. The comparison between the two groups with the paired *t*-test. GraphPad Prism software was used in statistical analysis. A *p*-value < 0.05 was considered statistically significant. * *p* < 0.05; ** *p* < 0.01.

**Figure 12:**
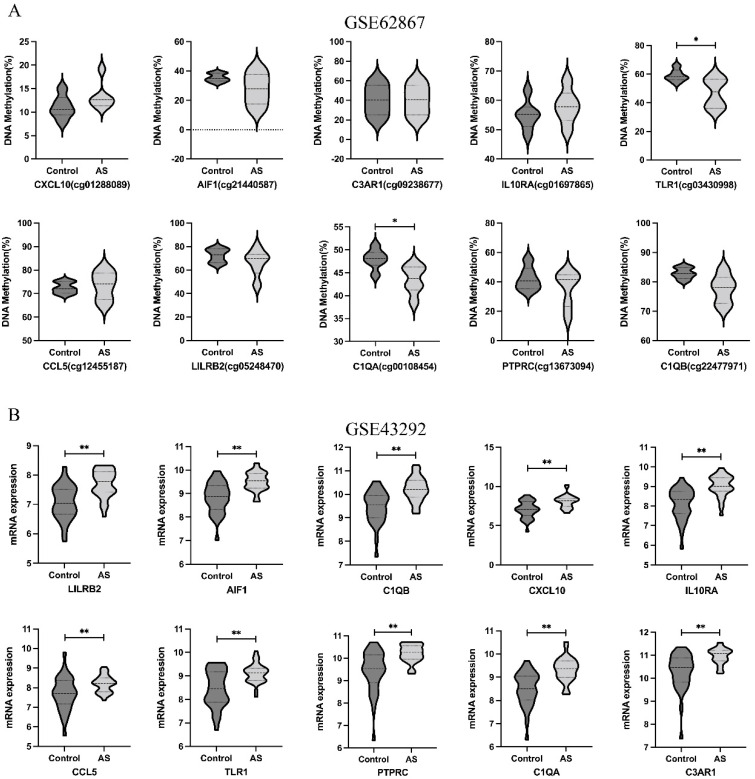
Validation of expression levels and methylation levels of hypo-up genes in AS in gene expression dataset (GSE43292) and DNA methylation dataset (GSE62867). (**A**) The methylation levels of hypo-up genes in GSE62867. (**B**) The expression levels of hypo-up genes in GSE43292. The comparison between the two groups with the paired *t*-test. GraphPad Prism software was used in statistical analysis. A *p*-value < 0.05 was considered statistically significant. * *p* < 0.05; ** *p* < 0.01.

We used the data from GSE62867 and GSE43292 to validate the methylation and expression levels of the hub genes, respectively. Following validation, ACTG2 and LMOD1 were confirmed to be hypermethylated and downregulated in atheroma plaque compared to normal arteries (Figure 11), while TLR1 and C1QA were hypomethylated and upregulated in atheroma plaque (Figure 12).

### 3.9. Validation of the Candidate Hub Genes in Human Peripheral Blood Samples

**Figure 13:**
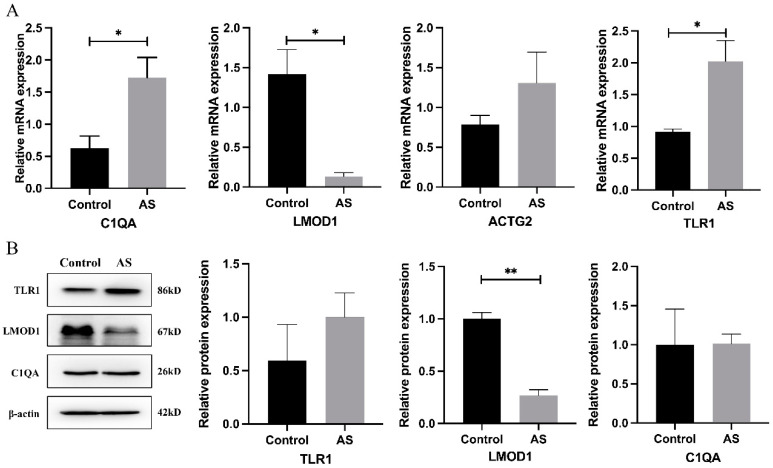
The mRNA levels and protein levels of candidate hub genes were detected in peripheral blood mononuclear cells (PBMCs) from arteriosclerotic patients and matched control subjects by RT-qPCR and Western blot analysis. (**A**) The mRNA levels of four candidate hub genes by RT-qPCR analysis (* *p* < 0.05). (**B**) The protein levels of three candidate hub genes by Western blotting, with relative protein quantification of Western blotting by densitometric analysis. * *p* < 0.05; ** *p* < 0.01.

We collected the PBMCs from atherosclerotic patients and healthy donors, and then extracted the mRNA to confirm the mRNA expression levels of candidate hub genes, including ACTG2, LMOD1, TLR1, and C1QA (Figure 13A). The results indicated that C1QA, LMOD1, and TLR1 were downregulated in PBMCs from atherosclerotic patients compared to those from healthy donors, and the difference was statistically significant. However, there was no significant difference in ACTG2 mRNA expression level between the two populations. Next, we detected the protein expression levels of C1QA, LMOD1, and TLR1 in PBMCs from the two populations (Figure 13B). The results showed that the protein level of LMOD1 in PBMCs was significantly downregulated in atherosclerotic patients compared to that of healthy donors, while there was no significant difference in C1QA and TLR1 protein levels between the two groups. 

### 3.10. The Methylation Levels of CpG Sites in LMOD1 Promoter

**Figure 14:**
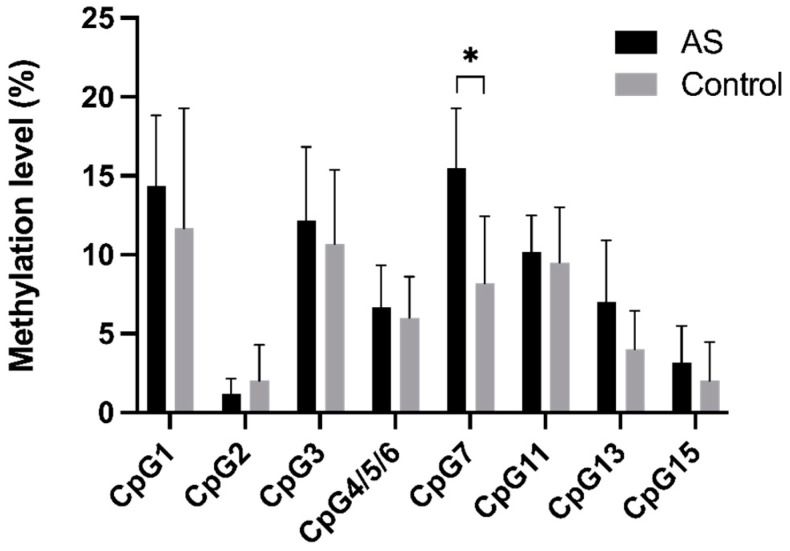
Comparison of methylation levels of 10 CpG sites (CpG1–7, 11, 13, and 15) at LMOD1 promoter in PBMCs from arteriosclerotic patients to the matched control subjects using the MassARRAY platform. * *p* < 0.05.

DNA methylation, especially in the promoter region, is a heritable epigenetic modification that plays a key role in the regulation of gene expression and phenotype modification. In the present study, the methylation levels of 10 CpG sites (CpG1–7, 11, 13, and 15) in the LMOD1 promoter were detected in six healthy controls and six atherosclerotic patients using the MassARRAY platform (Figure 14). All in all, the methylation levels of nine CpG sites (CpG1, 3–7, 11, 13, and 15) in the LMOD1 promoter were elevated in atherosclerotic patients except for one CpG site (CpG2). Compared to that of the healthy control group, the methylation level of CpG7 in the LMOD1 promoter was significantly elevated in atherosclerotic patients (15.5% ± 3.45% vs. 8.17% ± 3.89%, *p* = 0.0103). 

## 4. Discussion

Epigenetics has dramatically influenced our understanding of gene regulation in disease. The modifiable nature of epigenetic marks makes them key targets for future therapies. DNA methylation is a well-studied and stable form of epigenetics, and it has been shown that the alterations of DNA methylation are related to many diseases [14,15,16,17]. This has drawn increasing attention to the importance of methylation in the pathophysiology of AS. DNA methylation has been observed as an overlapping characteristic of AS and AS-related stimuli [18]. Studying the effect of methylation on the pathogenesis of AS is critical for gaining insights into the AS.

In this study, we firstly analyzed the DNA expression dataset. The results indicated that dysregulated genes were mainly associated with vascular smooth muscle contraction and immune-inflammatory activation. It is well known that the loss of vascular smooth muscle contractility contributes to hemodynamic changes, hypertension, and arterial stiffness, all of which have been implicated in the pathogenesis of AS. The activation of the immune-inflammatory response has been proven to participate in AS pathogenesis [19]. Interestingly, KEGG pathway analysis of upregulated genes in AS revealed genes mainly related to tuberculosis, phagosome, and osteoclast differentiation. Recent studies have demonstrated that there are close epidemiological and pathogenetic overlaps between tuberculosis and AS, and that tuberculosis is a nontraditional risk factor for AS [20,21]. Moreover, it has been revealed that the osteogenic transition of vascular smooth muscle cells (VSMCs) plays a key role in the formation of calcification in atherosclerotic lesions. In addition, following phagocytosis, VSMCs transform into foam cells after uptake of LDL, which plays a key role in the occurrence and development of AS. The artery signatures identified by our methods were consistent with AS pathological mechanism, indicating that these dysregulated genes are feasible as the biomarkers for AS.

In recent years, several studies reported the effect of aberrant DNA methylation on immune response and vascular function in AS development [22,23]. In our study, we analyzed genome-wide DNA methylation changes between normal artery tissue and atherosclerotic plaque lesions. GO term enrichment analysis found that hypermethylation was mainly involved in actin filament organization and actin binding, indicating that hypermethylation markedly affects arterial contractility, which was also discovered in KEGG pathway analysis, showing that hypermethylation was significantly associated with vascular smooth muscle contraction. Furthermore, enrichment analysis showed that DNA hypermethylation participated in cell junction and adhesion in AS, which has been demonstrated by previous studies [24,25]. On the other hand, our results discovered that hypomethylation was mainly related to the activation of T cells and neutrophils, which suggests that the immune-inflammatory response is regulated by hypomethylation in the pathogenesis of AS. In our study, the results of enrichment analysis of downregulated genes and hypermethylated genes were similar, all of which were markedly associated with vascular smooth muscle contractility. Likewise, the results of enrichment analysis of upregulated genes and hypomethylated genes were also alike, which were mainly related to activation of immune inflammation. These findings reveal that DNA methylation is a concomitant feature of AS pathogenesis, and the development of AS may be generally influenced by gene methylation.

In our study, hypo-up genes were primarily linked to the regulation and activation of immune-inflammatory activation, cytokine response, tuberculosis, and osteoclast differentiation. These results further confirmed that AS is a chronic inflammation and immune-related disease [26]. The hyper-down genes were mainly enriched in muscle system process, actin binding, and vascular smooth muscle contraction, which was consistent with the results of function enrichment of downregulated genes in the DNA expression dataset. These results revealed that VSMC contractility is highly correlated with the pathogenesis of AS. VSMCs express a unique repertoire of signaling molecules and contractile proteins required for VSMC contractile function, which are regulated and maintained by multiple contractile genes. The hyper-down hub genes screened in the present study are smooth muscle cell (SMC) contractile phenotype marker genes (LMOD1, MYH10, MYH11, CNN1, TAGLN, TPM2, MYOCD, and ACTG2), all of which are smooth muscle-restricted actin-binding proteins that are involved in the modulation of smooth muscle contraction [27,28,29]. MYOCD acts as a transcriptional coactivator of serum response factor (SRF) and regulates expression of smooth muscle-specific SRF-target genes, including SMTN, MYH11, TAGLN, CNN1, ACTG2, and LMOD1 [30,31]. MYL9 has also been revealed to rely on SRF and myocardin-related transcription factors and is necessary for VSMCs contractility and cytoskeletal dynamics [32,33]. Sustained expression of these genes is critical for maintaining the contractile phenotype and normal function of VSMCs. Up to 80% of the cells in advanced atherosclerotic plaques originate from VSMCs according to studies on animal models [34]. VSMCs undergoing phenotypic transitions play a key role in the pathogenesis of AS. VSMCs suffering phenotypic transformation accumulate lipids, produce excessive extracellular matrix, undergo apoptosis, secrete proinflammatory cytokines and growth factors, and lead to monocyte recruitment [35]. Furthermore, there is recent evidence showing that VSMCs undergoing phenotypic transformation can differentiate into macrophages and mesenchymal stem cells, resulting in lesion expansion and instability [36].

LMOD1, an SMC-specific gene, is regulated by the transcription of MYOCD. LMOD1 participates in SMC contractile activity and actin cytoskeleton assembly by binding to promyosin. In a previous study, LMOD1 was identified as one of 15 new potential risk loci in the pathogenesis of coronary artery disease [37]. There is increasing evidence that LMOD1 plays a critical role in the maintenance of differentiation and contractile function of VSMCs [38]. Levula et al. examined gene expression in plaque fragments derived from femoral arteries, carotid arteries, and abdominal aortic arteries; they found that the expression of LMOD1 in these samples was 6.5 times lower than that in normal control internal mammary arteries [39]. LMOD1 downregulation during AS might represent a crucial step in VSMCs phenotypic transitions. Decreased expression of LMOD1 also leads to increased migration and proliferation of VSMCs from the vascular membrane, contributing to the formation of atherosclerotic lesions, AS progression, and instability of plaque. In addition, LMOD1 was found to be highly expressed in the fibrous cap of atherosclerotic plaques, suggesting that LMOD1 may serve as a protective factor in AS, and help stabilize the developing lesions.

The effect of methylation on gene expression is dependent on its location within the genomic sequence. Therefore, locus-specific methylation might be a more reliable way to determine the relationship between DNA methylation and gene expression and health-related outcomes. Tissue-specific promoter hypermethylation has a significant effect on gene expression, especially in the CpG-rich promoter region. In a recent study, researchers found that DNA methylation regulated VSMCs contractility by influencing the promoter activities of smooth muscle-specific contractile genes (such as SM22α and SMA) and resulted in arterial stiffness associated with AS [40]. Zhu et al. demonstrated that atorvastatin, as the major class of drugs used to treat atherosclerotic cardiovascular disease, inhibits VSMCs proliferation and migration in AS, and this process is regulated by affecting DNA methylation in the p16 promoter region [41]. The aberrant methylation in the promoter region of MYH11 and CNN1 genes, two hyper-down hub genes screened in our study, was found to be associated with VSMCs differentiation in arterial disease [42]. In the present study, we found that the methylation levels of most CpG sites in LMOD1 promoter were elevated in AS. We speculate that the hypermethylation in LMOD1 promoter region may be related to the regulation of VSMC contraction and differentiation, which requires further experimental validation.

Despite the rigorous bioinformation analysis performed in the present study, there are still a few limitations. First, as a result of the relatively small sample size in this study, it might be possible to improve the reliability of the analysis results by expanding the sample size. Second, due to a lack of in vivo studies in this study, further research is necessary to determine the molecular mechanism of LMOD1 in the pathogenesis of AS. Third, guided by the bioinformatics analysis, our study initially revealed that a high methylation level in LMOD1 promoter might influence LMOD1 expression in AS, and future experiments are required to explore the potential mechanism of LMOD1 promoter methylation underlying the expression of LMOD1 and its effect on arteriosclerosis.

## 5. Conclusions

Our study suggests the potential use of methylation markers in AS diagnosis, and it may assist in the development of new epigenetic therapies. DNA hypermethylation affects AS formation mainly by regulating SMC-specific contractile phenotype genes that play a key role in VSMCs contraction and phenotype switching. LMOD1 exhibits potential as a promising diagnostic and therapeutic biomarker for AS. Nevertheless, further research is required to provide details on the underlying molecular mechanisms of the role of hypermethylation in AS development.

## Figures and Tables

**Table 1:** Sequences used for RT-qPCR.

Gene		Primer Sequences
ACTG2	Forward	CTCAAATACCCCATTGAACACG
	Reverse	TCAAGTGTTCATTGCTTTCTGG
C1QA	Forward	GGCTACTACTACTTCACCTTCC
	Reverse	TGGTAAATGTGACCCTTTTTGG
LMOD1	Forward	TCTAGAGTAGCCAAATATCGCC
	Reverse	TTTTCACAGAAGTTGAGCATGG
TLR1	Forward	CTGCAACATAACTCTGCTGATC
	Reverse	TTCTTCACCCAGAAAGAATCGT
β-actin	Forward	ACAGAGCCTCGCCTTTGC
	Reverse	CCACCATCACGCCCTGG

## Data Availability

Not applicable.
